# Enhancement of visual cues to self-motion during a visual/vestibular conflict

**DOI:** 10.1371/journal.pone.0282975

**Published:** 2023-03-15

**Authors:** Meaghan McManus, Laurence R. Harris

**Affiliations:** 1 Experimental Psychology, Justus Liebig University Giessen, Giessen, Hessen, Germany; 2 Centre for Vision Research, York University, Toronto, Ontario, Canada; Tokai University, JAPAN

## Abstract

Perceiving our orientation and motion requires sensory information provided by vision, our body and acceleration. Normally, these cues are redundant however in some situations they can conflict. Here, we created a visual-vestibular conflict by simulating a body-upright virtual world while participants were either standing (no conflict), supine or prone (conflict) and assessed the perception of “forward” distance travelled induced by visual motion. Some participants felt they were standing upright even when lying, indicating a visual reorientation illusion (VRI). We previously showed that when experiencing a VRI, visually induced self-motion is enhanced. Here, we determined if there was a relationship between VRI vulnerability and sensory weighting. Confirming our previous findings, the VRI-vulnerable group showed enhanced self-motion perception. We then assessed the relative weightings of visual and non-visual cues in VRI-vulnerable and VRI-resistant individuals using the Oriented Character Recognition Test. Surprisingly, VRI-vulnerable individuals weighted visual cues less and gravity cues more compared to VRI-resistant individuals. These findings are in line with robust integration where, when the difference between two cues is large, the discrepant cue (here gravity) is ignored. Ignoring the gravity cue then leads to relatively more emphasis being placed on visual information and thus a higher gain.

## Introduction

In our day-to-day lives we use a variety of cues to determine which way is up, including gravity, pressure from the support surfaces [1] and visual cues (see [2] for a review), as well as an internal prior that assumes our body is always upright: Mittelstaedt’s “idiothetic vector” [3]. These cues are integrated to provide a single overall estimate of the direction of up according to Bayesian principles [4, 5] in which they are assigned weightings according to their reliabilities [6, 7]. Causal inference may also influence the weightings given to the cues [5, 8, 9]. Although normally the various cues to self-orientation agree and are redundant, in some environments they may not always indicate the same direction. For example, when underwater the local horizon suggested by the structure of the ocean floor may not be orthogonal to gravity and when lying down the idiothetic vector is no longer aligned with gravity. In such situations placing too much reliability on one or other cue can lead to dangerous errors in perceived orientation. In the even more extreme environment of space, vestibular and somatosensory cues to self-orientation are absent leading to an arbitrary personal definition of up that is subject to sudden change: a visual reorientation illusion (VRI, [10, 11]). Virtual reality can be used to created VRIs on Earth since any amount of disagreement between visual and non-visual cues can be created by tilting the virtual environment such that the visual up does not agree with the gravitational up. People vary in their vulnerability to virtual-reality-induced VRIs [12] and also in the weightings they apply to the various sources of information that contribute to their perceived orientation [4, 13, 14]. We previously found that individuals who report VRIs also show an enhancement in their perception of optic flow compared to those who do not report VRIs [12]. In McManus and Harris [12] we put forward a model which hypothesized that the enhancement of optic flow during a VRI might be due to an increase in weight given to visual cues when the visual up is misaligned from the gravitational up. Here we use virtual reality to induce a VRI and determine the relationship between people’s vulnerability to VRI’s with the relative weightings they give to visual and non-visual orientation cues and test this hypothesis.

**Fig 1:**
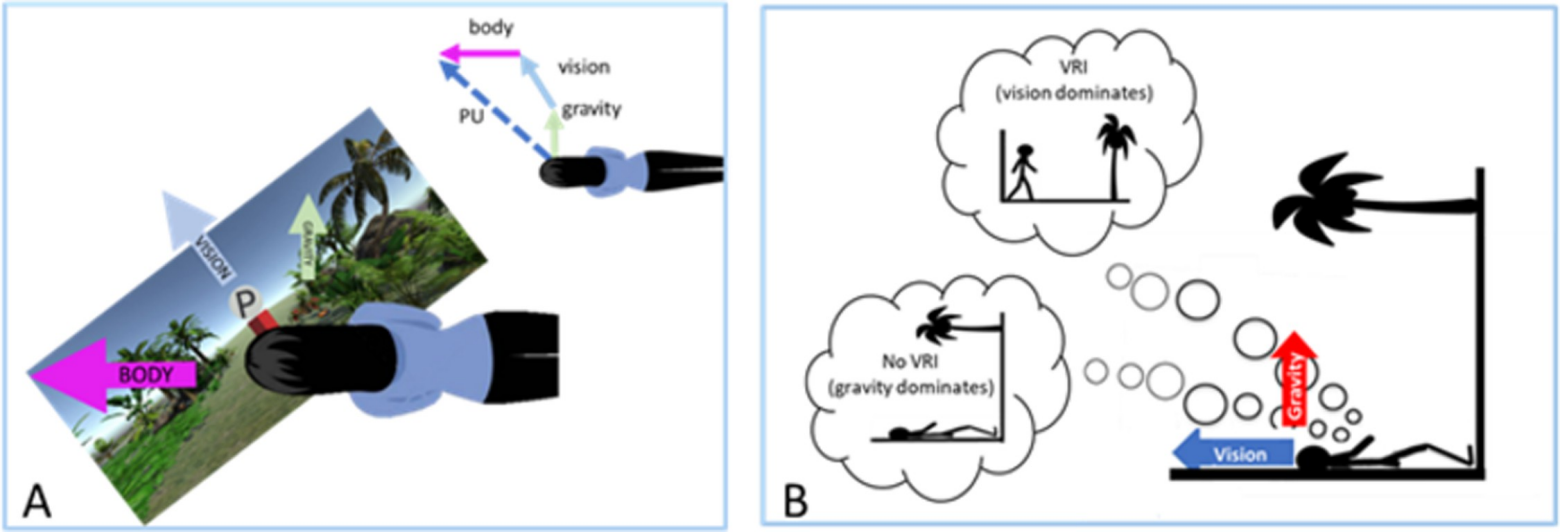
Determining upright. Both panels depict different ways a person might determine where “up” is. Panel A illustrates how the cues to “up” can be separated into different directions. In this case, a person is lying on their left side while viewing a visual environment tilted at 45 degrees. By measuring the PU as these orientations are varied, the relative strengths of their various contributions can be calculated by simple vector geometry as show in the inset. Panel B provides an example of a visual reorientation illusion (VRI). A person is lying on their back in a room that has been tilted such that the visually signalled direction of up aligns with their body orientation. The visual cues in the environment (blue arrow) indicate that the person is upright while the up denoted by gravity (red arrow) signals that the person is lying down. Reliance on the blue arrow results in a VRI while reliance on the red arrow results in no VRI.

There are multiple ways of assessing the perception of up, including the subjective haptic vertical (SHV [15]) the subjective visual vertical (SVV [16, 17]) and the perceptual upright (PU [4]). Curiously, these different methods of assessing perceived orientation do not always agree [18] suggesting that the contributions of the various cues may differ depending on the task at hand. For instance, the SVV is based on imagining the direction in which a ball would fall and therefore cannot provide an estimate of “up” in microgravity [19]. In contrast, the perceptual upright (PU), the orientation in which things look most upright and are most recognizable [4, 20], is still present as an “up” even without gravity [19]. The PU can be measured using the oriented character recognition test (OChaRT [4]). Participants view an ambiguous symbol “P” in various orientations and indicate whether it looks more like a p or more like a d. The OChaRT finds the two most ambiguous orientations where participants are equally likely to see a p or a d, the bisector of which is taken as the PU. By varying the relative orientations of the main component cues (visual, gravity and the body), for example by having participants stand and lie on their side, and varying the orientation of the visual background, the relative contributions of vision, the body, and gravity can be determined by simple vector geometry (see Fig 1A). Typically, vision accounts for about 25% - 36% of the cue contribution to the PU, the body about 52–54% and gravity about 10–20%, however large individual differences are seen [4, 19].

Howard and Hu [21] found that when supine in a fully furnished room that was rotated such that visual up aligned with the participants’ longitudinal body axis, 84% of individuals felt that they were actually standing upright i.e., they experienced a Visual Reorientation Illusion (VRI) [16, 21–23]. The VRI concept is shown diagrammatically in Fig 1B. In a strange simulated visual environment with palm trees growing horizontally out of the wall there is a conflict between gravity and the direction of “up” indicated by visual information. It appears that most people resolve this conflict within the complete immersion provided by a full-field room visually and ignore or put much less weight on the gravity vector [21] leading to a VRI.

When participants experience a VRI on Earth (e.g., they are actually lying down but feel they are standing), they require less visual motion (optic flow) to create the illusory sensation of moving through a given distance in virtual reality than when they are physically standing in an upright world [12]. Requiring less visual motion to evoke the sensation of travelling a given distance suggests an increased sensitivity to optic flow information. Thus, both their perception of orientation and their greater response to optic flow imply that vision is the more powerful cue for participants vulnerable to experiencing VRIs compared to individuals who do not report a VRI. To confirm this, here we measured the relative weighting for visual, body, and gravity cues in people who were or were not susceptible to VRIs. We performed two experiments: first, a move-to-target self-motion task (MTT) with the participants in different orientations (but with visual orientation cues always aligned with their body) to assess the effectiveness of optic flow and second, we used the OChaRT to assess the relative weightings of the cues determining the PU (after [4]). Participants were divided into two groups according to their vulnerability to experiencing VRIs. We hypothesized that individuals who were more vulnerable to a VRI, our “VRI-vulnerable” group, would require less visual motion to travel through a given distance in virtual reality compared to those who did not report a VRI, our “VRI-resistant” group. We further hypothesized that VRI-vulnerable individuals would rely more on vision when determining the PU than the VRI-resistant individuals as indicated by an increased relative visual weighting. The VRI-vulnerable group should also have a lower relative weight given to the gravity cue compared to the VRI-resistant group. Understanding how differences in sensory weighting might be related to differences in the perception of things as fundamental as perceived orientation and self-motion have important implications for many fields of inquiry from research in unusual environments such as microgravity [24], aging [25], heading [26], and motion sickness [14], as well as important implications for use of virtual reality technology.

## Methods

### Participants

The experiment had 41 participants (mean age 21.05 ± 4.33 yrs, 25 women). This study conformed to the standards of the Canadian Tri-Council Research Ethics guidelines. The experiment was conducted in agreement with the Declaration of Helsinki (1964) and was approved by the Human Participants Review Sub-Committee of York University’s Ethics Review Board (Certificate # e2017–111). Before taking part in the experiment, all participants provided written consent after reading the informed consent forms. All participants were naïve as to the purpose of the study at the time of testing. They reported normal or corrected-to-normal vision and no vestibular, balance, or depth perception problems.

### Apparatus

Stimuli were presented in an Oculus Rift CV1 virtual headset. The CV1 has a field of view that extends approximately ±110° diagonally. The screen has a 1,080 x 1,200-pixel resolution per eye and a 90Hz refresh rate. Stimuli were created in Unity (Version 5.5.2f1, Unity Technologies SF, US) on an Alienware Area-51 R2 with an intel i7 core, and a Nvidia GeForce GTX 980 graphics card. The projection was stereoscopic and was actively linked to the position of the participant’s head. Therefore, distance cues were available from stereoscopic cues and motion parallax.

### Visual stimuli

#### Move-to-Target task stimuli

**Fig 2:**
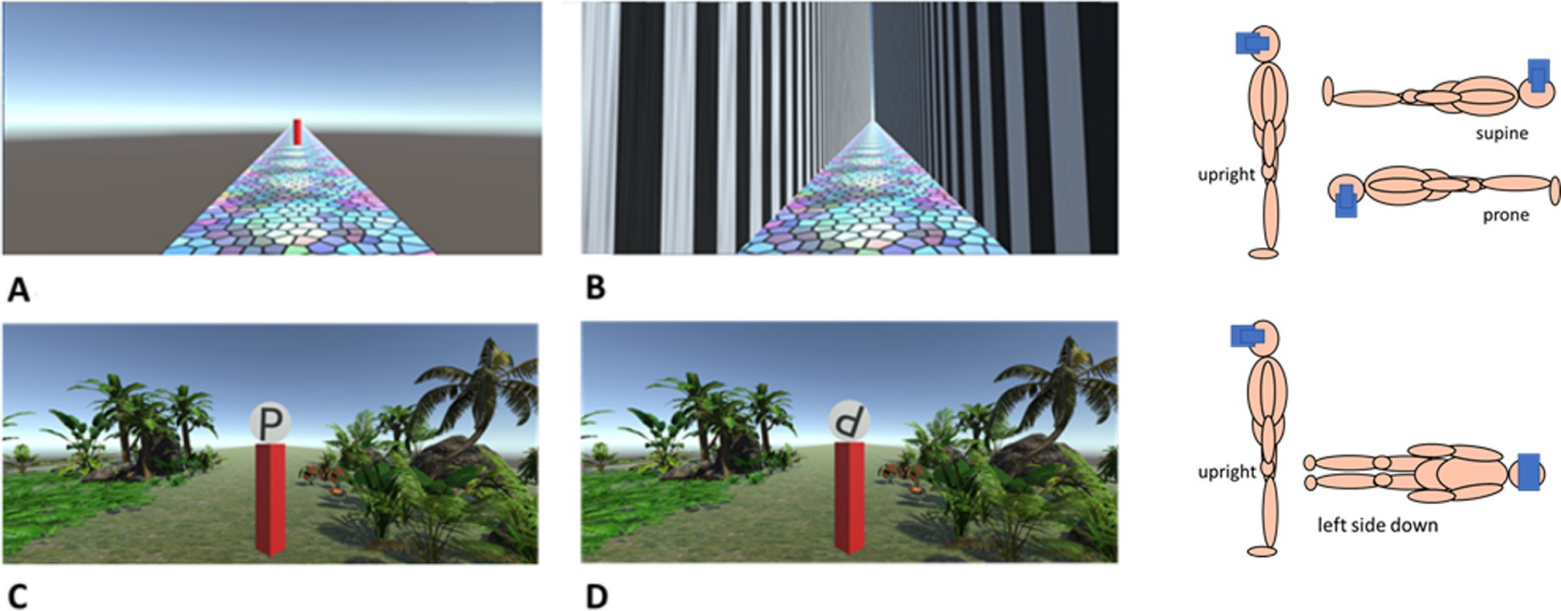
Screen captures of the different environments used in this experiment. (A) is the hallway shown while the target is visible and the walls are invisible, and (B) is the hallway once the participant has clicked the left mouse button and they are beginning to move. (C) is an example of the upright (0 degree) visual scene in the OChaRT environment. (D) Is the same visual scene as C but with the ambiguous p/d shape at a different orientation. The cartoons on the right-hand side of the image show the different body postures that were used when completing the different tasks in each environment. The people are wearing HMDs. In the hallway environment depicted in panels A and B, the upright, supine, and prone postures were used. In the OChaRT environment depicted in panels C and D, the upright and left side down postures were used.

The virtual hallway consisted of a multicolored non-reflective stained-glass floor with a simulated width of 9m. There were walls on either side of the floor that had vertical black and white stripes (each stripe was approximately 1m wide) and were 1,000m high so that the viewer could not see over the top (see Fig 2B). The walls and the floor extended 10,000m in front of the viewer. There was no ceiling, so the simulated blue sky and sun provided the light source. In additional there were no shadows in the environment. The horizon was at eye level so that participants could see down the hallway and was also present when the walls were not visible (Fig 2A).

The target distance was indicated with a red 3D rectangular box 5m x 1m x 1m in size (see Fig 2A) drawn with one edge towards the viewer. The hallway’s walls were not visible at the same time as the target was presented to prevent lining it up with some feature on the walls. The distance to the target was defined as being from the center of the participant (the location of the camera) to the center of the target. Participants’ eye height was set to the top of the pillar so that the viewing angle of the scene was the same for all participants.

#### OChaRT environment stimuli

The virtual environment consisted of a grass floor surface with several types of virtual plants, rocks and motionless virtual frogs. In the centre of the environment was the same red target pillar as used in the MTT environment. It was presented at a simulated distance of 10m from the participant and had the same size and orientation as the pillar in the MTT environment. Resting on top of the red target was a flat white disk with a diameter of 2m. In the center of the disk was an ambiguous symbol “P” (Fig 2C). Illumination was from the same location and direction as in the hallway environment and the horizon was presented at eye height.

### Tasks

#### Overview

Participants first completed a virtual reality Move-To-Target task where they moved to different distances indicated by a pillar (Fig 2A). They performed the task in three different postures. After each posture they verbally completed a questionnaire to help determine how they perceived their orientation during the task. Afterwards they did a timed task to determine how consistent their VRI experience was. Following that, participants completed the OChaRT task where they were in a different virtual reality environment (see Fig 2C and 2D) and reported if a letter presented in front of them in the environment looked more like the letter p or the letter d. On each trial, the background orientation and the orientation of the letter changed. Participants completed the task in two postures. After the task they verbally reported if they had experienced a VRI during the task.

#### Move-to-Target Task (MTT)

At the beginning of every trial, participants saw the target (Fig 2A) projected at a pseudo-randomly determined simulated distance of either 10, 20, 40, 60, or 80m. Participants were instructed to pay attention to how far the target was from them and, when ready, to click the left mouse button. Immediately upon the click, the target disappeared, the hallway walls appeared, and the participant began to virtually accelerate at 9.8m/s^2^ down the hallway. When the participant felt that they had reached the location of the previously visible target (i.e., when their head was inside the now-invisible target) they clicked the right mouse button to stop the motion. Immediately afterwards the next trial started with the participant repositioned at their original position in the hallway with the target at a new distance from them and the walls rendered invisible. Each target distance was presented to each participant 10 times resulting in 50 trials per participant. Two additional trials, one at the beginning of the experiment with the target at 5m and one at the end with the target at 200m were also included but were not used in the analysis. The first was used to familiarize the participant with the environment and how the trials worked and in the last trial the far target indicated that the experiment was over.

Participants completed the task three times: standing upright, lying supine on a bed with their feet against a wall, and lying prone on a bed with their head off the edge of the bed. In the prone posture a box was placed beneath the person’s feet to simulate the wall. The order of body orientations was determined using a Latin square design.

#### OChaRT task

The participant’s task was to decide if an ambiguous symbol P (Fig 2C and 2D), presented in various orientations, looked more like a “p” or more like a “d”. Responses were forced choice and were made with mouse clicks: a left click indicated a “p” and a right click indicated a “d”. The stimuli were presented for 500 milliseconds at which point the screen went black and stayed black until a response was made. The angle of the symbol was varied in the subsequent trial based on the participant’s response following a Parameter Estimation by Sequential Testing (PEST) function [27]. The PEST finds the two points of subjective equality (PSE) where the character is equally likely to be identified as a “p” or a “d”.

Participants completed the task once lying on their left side and once standing, with the order varying by participant. In the left-side-down posture, layers of foam were placed under the participant’s head so that it would be level. For each of the two body postures there were five visual angles of the visual environment for a total of 10. While standing the visual angles were 0°, 40°, -40°, 90°, and -90°, in gravity centered coordinates. While lying with their left side down the visual angles were -110°, 20°, -30°, -70°, and -160° in gravity centered coordinates. For each of the five visual background angles there were two ranges of orientations of the character, one that started with the character at 0° (an upright ‘p’) relative to the visual environment, and one that started at 180° (a ‘d’) relative to the visual environment. For the starting points, a random angle between +45° and -45° (where positive indicates tilted left relative to upright in the visual environment) was added. There were thus 10 staircases per body posture, which were interleaved such that on each trial one of the 10 staircases and its corresponding background would be randomly selected but could not be selected again until all ten had had a trial. Each body posture took approximately 10-15mins to complete.

#### VRI measures

During the OChaRT, the visual background changed every trial so a VRI could not be determined at the end of each body posture as accurately as the MTT. As well, the VRI likelihood while left-side-down might not be the same as the likelihood of experiencing a VRI while supine or prone. Therefore, participants were grouped based on the overall likelihood that they experienced VRIs over the course of the experiment.

VRI experience was measured in three different ways over the course of the experiment to determine participants’ VRI vulnerability. Participants completed a questionnaire as per Experiment 3 in McManus and Harris (2021) (VRI MTT measure, see S1 File) following each body posture of the MTT which determined if they had experienced any VRIs during the MTT. The participant listened to four options describing what they might have experienced in the VR environment during the MTT and selected which one they felt best matched their experience. If they selected the option that indicate that they had felt upright and moving forward, they were grouped as having a VRI. They also completed a timed VRI task while in the MTT environment which measured the consistency of the VRI over the course of approximately 1.5 seconds. They indicated the presence of a VRI by pressing and holding the left mouse button and the right mouse button to indicate they were not (VRI Over Time measure). Lastly, following each posture of the OChaRT, they reported whether or not they felt they had had a VRI during the previous trials (VRI verbal report). Based on their responses to the three tasks participants were grouped as being “VRI-vulnerable”, “VRI-resistant”, or they were grouped as “Other”. See the S1 File section “VRI Measurements” for a detailed description of the different measures and the grouping criteria.

#### General procedure

After reading and signing the consent form, participants were shown a letter p and a letter d and had to verbally indicate what they saw. All participants could correctly identify the letters.

Participants were then shown how to put on the HMD and how to adjust it using the straps on the side and top of the headset so that the visual environment was clear. They then adopted the appropriate first MTT posture and ran through the MTT task (see above). Afterwards they removed the HMD and completed the VRI MTT measure. The MTT task was then repeated in the 2^nd^ and 3^rd^ body postures with the VRI MTT measured after each posture. In all, the three postures, explanations, and VRI measures took approximately 30 minutes.

Once the MTT task was complete, the concept of a VRI was explained to participants in detail with examples. Participants were told that some people experience the illusion often, some people experience it some of the time, and some people never do, and that each type of person was valuable to the experiment. Participants were told their data would be analyzed based on which VRI group they were in, so it was important they answer honestly. This was done to try to reduce any “good participant” bias where the participant might claim to experience a VRI when they did not. Once we felt participants understood the concept, participants put the HMD back on and completed the VRI Over Time measure. In all, including explanation, this task took approximately 10 minutes.

After the VRI Over Time measure was completed, participants removed the HMD. They were then placed in either a standing posture or the left side down posture and the instructions for the OChaRT were explained. Once the headset was back on, the OChaRT task was started for that posture. Upon completion participants removed the HMD, were put into the next posture and completed OChaRT in that posture. In all, with explanation, the OChaRT task took 20 minutes in total. After the left-side-down OChaRT posture, the VRI verbal report was completed and recorded by the experimenter. Following the OChaRT participants were debriefed, and any questions were answered. Overall, the entire experiment took about 65minutes.

## Data analysis

### Moving to target

Once the MTT data were collected, the average distance traveled was found for each target distance in each posture for each participant, resulting in 15 means for each participant. The S1 File under the “MTT Outlier Analyses and Distribution of Data” heading contains the outlier analysis that was performed as well as the distribution of the data.

Participants were then divided into the three VRI groups described in the VRI Measures above. Four participants were in the “Other” group and their data were not used in the rest of the analysis. Then, for each person, the gains of the travel distances for each target distance were calculated for each posture. The gain is defined as perceived distance (the target distance) expressed as a fraction of the actual distance travelled and was used as a measure of the effectiveness of the optic flow cue in eliciting the perception of motion. Gains less than 1 indicate that participants had to travel further than the target distance to feel they had passed through the target distance (less effective use of visual cues to motion, low gain) and vice versa.

### Modeling the contribution of vision, the body, and gravity on the PU

For the OChaRT, the midpoint between the two PSEs was found. This resulted in 10 estimates of the PU for each participant (2 postures and 5 background orientations). Reported angles are in body coordinates, with 0° being above the head, and negative numbers counterclockwise. See the S1 File section “OCHaRT Outlier Analysis” for the outlier analysis that was done for the OCHaRT data.

For each participant, we modeled the relative contributions of the visual, body and gravity cues (see Fig 1A) in determining the PU using a weighted vector sum model (see 4), where the length of each vector corresponds to its relative weight, by fitting the following formulae to each person’s data set.


$$\vec{PU}=\vec{vision}.w_{v}+\vec{gravity.}w_{g}+\vec{body.}w_{b}$$ Eq (1)

$${\text{W}}_{\text{v}}+{\text{W}}_{\text{g}}+{\text{W}}_{\text{b}}=1$$ Eq (2)

Where w_v_, w_g_, and w_b_ correspond to the relative weights assigned to the visual, gravity and body vectors respectively. The model was fit to each person’s data using the Marquardt-Levenberg technique [28]. The model was constrained such that a negative weight was set to 0.

## Results

### VRI group

Overall, 51% of participants were classified as VRI-vulnerable and 39% were found to be VRI-resistant. The other 10% were classified as “Other” and were excluded from the analysis. For a breakdown of the VRI measures, see “Breakdown of VRI Measures” in the S1 File.

### Move-to-target

#### Gain by VRI group

A linear mixed model was used to compare the effects of VRI group (2), target distance (10), and posture (3) on travel distance in the virtual reality environment. Posture and target distance were set as fixed repeated effects, VRI was set as a fixed effect, and the participant ID was set as a random effect (random intercept) to help take into account the variability in performance between participants. Degrees of freedom were approximated using the Satterthwaite method [29]. Post hoc analyses were made using the Sidak correction.

**Fig 3:**
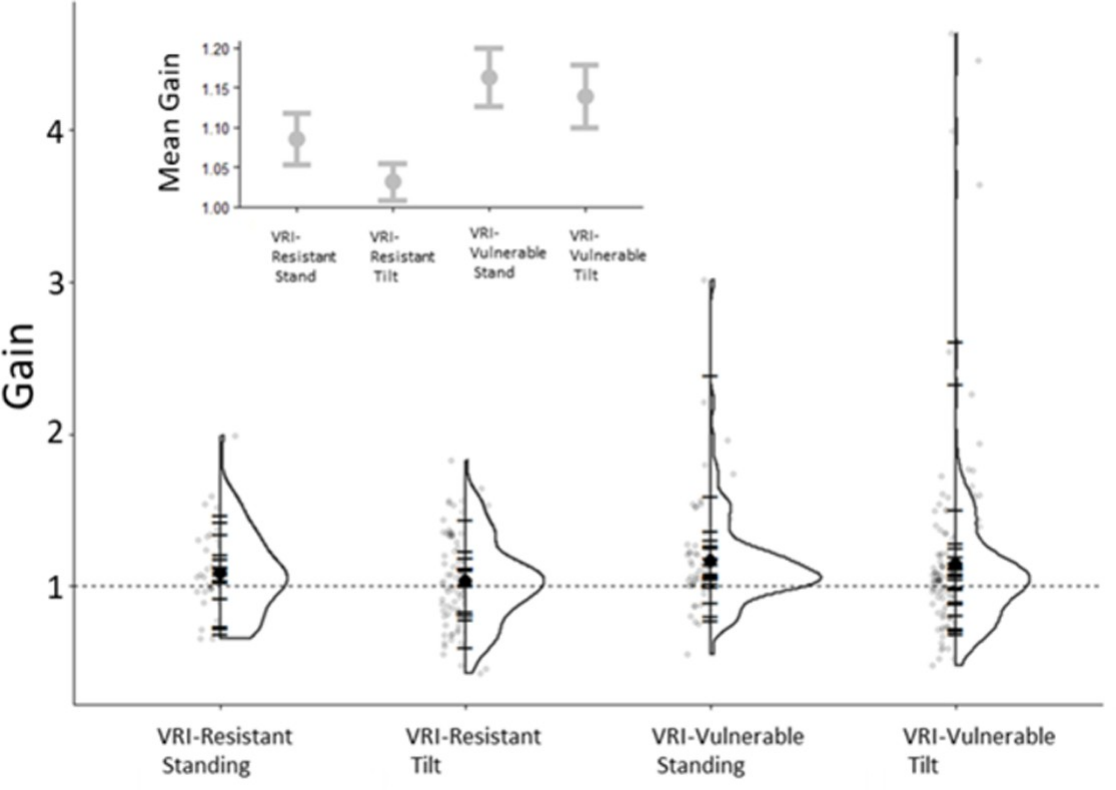
Gain by VRI type. The average gain for each participant is plotted as half violin plots where the width of the area under the line represents the proportion of data that is plotted there. The black horizontal lines display the means for each participant while the grey dots are individual data points. The horizontal dashed line shows unity gain (perfect performance). The insert shows the mean gain for each condition and the corresponding standard error.

A main effect of VRI group on gain was found *F*(1, 248.647) = 7.728, *p*< 0.006. The mean gain for the VRI-vulnerable group was 1.15 (SE = 0.024) where the gain while standing for the VRI-vulnerable standing group was 1.16 (SE = 0.036) and while tilted it was 1.14 (SE = 0.033). The mean gain for the VRI-resistant group was 1.06 (SE = 0.027) where the gain while standing for the VRI- resistant standing group was 1.09 (SE = 0.032) and while tilted it was 1.03 (SE = 0.022). A main effect of distance on gain was also found *F*(4, 136.434) = 3.962, *p*< 0.004. No other effects were found. The average gains for each VRI group and the gains for each participant at each VRI group are displayed in Fig 3.

### Perceptual upright and VRI vulnerability

**Fig 4:**
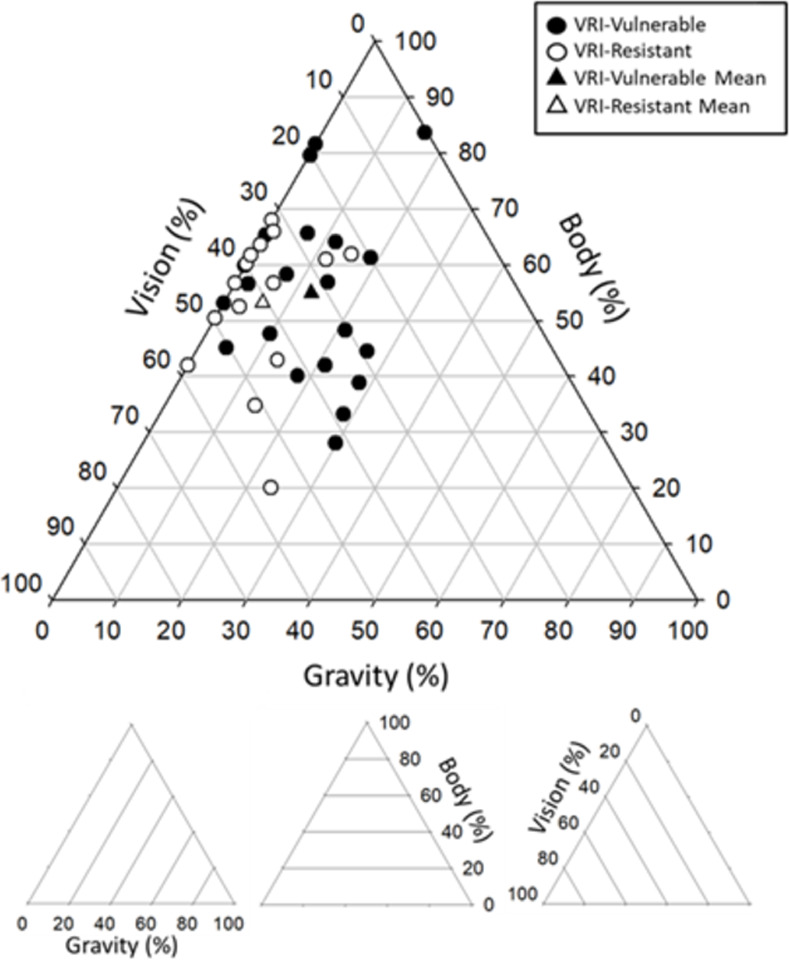
The relative weighting of vision, the body and gravity for participants in the VRI-vulnerable (filled circles) and VRI-resistant (open circles) groups are plotted as a ternary plot. Means are shown as filled (VRI-vulnerable) and open triangles (VRI-resistant). The inset below the plot is a key indicating how to read a ternary plot. The gravity weightings are read along the diagonal lines starting at the bottom left of the triangle where 0% gravity weighting corresponds to the left-most diagonal line. The body weights are read along the horizontal lines with 0% body weighting corresponding to the bottom-most horizontal line. The visual weights are read along the diagonal lines starting at the apex of the triangle where 0% visual weight corresponds to the right-most diagonal line.

Data were fitted with the three-vector model as described in the methods (Eq 1) to calculate the relative weights of vision, the body and gravity for each participant. To compare our two groups, we ran three separate 2 tailed t-tests with heterogenous variance (Welch’s t-test) on these weightings with alpha set at 0.05. Gravity was given a significantly higher weighting and vision a significantly lower weighting in the VRI-vulnerable group compared to the VRI-resistant group. The results are shown in Table 1 and the weightings for each participant are plotted as a ternary plot in Fig 4 for both the VRI-vulnerable (filled circles) and the VRI-resistant (open circles) groups.

**Table 1:** The results of the three t-tests comparing sensory weight between the VRI-vulnerable and VRI-resistant groups. The average relative contribution of each cue in determining the PU is given as a percentage. The numbers in brackets are the standard deviation. The columns are the three cues, the rows are the VRI group. The alpha denotes the significance value from the t-test, with the bolded alphas indicating a result with a p less than 0.05.

Mean Weight (SD)	Gravity %	Body %	Vision %
**VRI-vulnerable**	12.66 (±10.59%)	54.91 (±15.28%)	32.41 (±11.53%)
**VRI-resistant**	5.95 (±7.76%)	53.22 (±13.22%)	40.82 (±10.16%)
**T- test (alpha)**	***t*(33.96) = 2.19,**	*t*(32.66) = 0.35,	***t*(31.19) = -2.31,**
	***p* = 0.035, *d* = 0.70**	*p* = 0.723	***p* = 0.027, *d* = -0.77**

## Discussion

When people experience conflicting frames of reference that indicate lying and standing at the same time, some are dominated by the visual cue and are said to experience a visual reorientation illusion (VRI). Others are more aware that they are lying down and do not experience a VRI. Here, we confirm our earlier work [12] showing that a person who is likely experiencing a VRI (i.e., lying down but feeling they are standing up) needs less visual motion (higher gain) to feel that they have travelled through a given distance than a person who is less likely to experience a VRI. These results are also in line with [30] where participants experienced enhanced self-rotation during a VRI. In the present experiment our VRI-vulnerable group needed to travel approximately 8.36% less far through a virtual environment to perceive that they had passed through a particular distance than VRI-resistant individuals (Fig 3).

The enhanced gain during the MTT for the VRI-vulnerable compared to the VRI-resistant group initially seemed to suggest an enhancement in the processing of visual information during a VRI which we hypothesised might correspond to such individuals placing a higher weighting on vision in the multisensory process of determining their motion and orientation [12, 21, 30]. Indeed, we did find significant differences in the relative weightings given to the visual, body, and gravity cues for determining perceived orientation in individuals who were more likely to experience a VRI compared to those who were less likely to experience a VRI. Interestingly however, and contrary to our hypothesis, members of the VRI-vulnerable group relied relatively more heavily on the gravity cue compared to the VRI-resistant group and also relied relatively less on vision compared to the VRI-resistant group when determining the perceptual upright (Fig 4 and Table 1). This seems counterintuitive, why might it be?

### Conflict detection and cue weighting

We have previously found that environments with strong conflicting visual and gravity information result in an increase in experiencing a VRI [12].

In situations where there is a conflict between gravity and the visual upright, individuals who are more likely to experience VRIs would be those more sensitive to a conflict between the visual and gravity uprights. In the current experiment, the individuals most likely to report VRIs during the MTT were those with a higher gravity weighting. They may therefore be more sensitive in detecting a visual-vestibular conflict between the orientation cues which in turn would then affect the perceived reliability of the cues [5].

In the current study, our participants had normally-functioning vestibular systems, so like the control participants in Dakin et al. [31], they would experience conflict between visual and vestibular cues during the MTT. This is relevant here because participants who weighted the gravity cue more would experience a more intense visual-vestibular conflict here than those who weighted gravity less. In fact, many of the VRI-resistant individuals had a gravity weighting of 0% and would likely not have noticed any conflict. We had hypothesized that the higher self-motion gain in VRI-vulnerable people corresponded to them placing a higher weighting on vision [12, 21, 30]. But then why, after detecting a conflict, would individuals who weight gravity higher then go on use vision more effectively?

### Robustness

According to Bayesian integration theory, during multisensory integration information from each sense is weighted depending on its reliability. That is, the more reliable or less noisy a cue is, the higher it is weighted [6]. This would suggest that for the weights given by the vector sum model, the cue with the lowest variance should have the highest relative weight. Unfortunately, we were not able to measure each of the variances for gravity, the body, and vision separately as the body cue is always present, as explained in Dyde et al. [4], but, following Bayesian integration principles, we can infer from the higher weighting that VRI-vulnerable individuals applied to the gravity cues that that cue would have a higher internal precision for them compared to VRI-resistant individuals [4, 6, 32].

Integrating cues works well to improve the reliability of the overall estimate when the difference between the information provided by each cue is small. But sometimes cues can give very different estimates, such as the different uprights of the ambiguous hallway environment viewed while lying down in the current experiment. When the difference between the two estimates is large, a person might exhibit robust integration where the cue that is more discrepant is discounted or “vetoed” [6, 33–35]. Such robust integration can lead to changes in behaviour [6, 36]. In their review on causal inference, Shams and Beierholm [9] discuss reports that animals that navigate using both path integration and landmarks give a larger weight to landmark cues if the two cues provide similar estimates. However, if there were a large discrepancy between the information provided by path integration and landmarks, then despite its higher weighting, the landmark cue is ignored, and the animal relies on path integration. But how is the discrepant cue determined? How would people know which cue to veto?

In the current experiment during the MTT the body up is aligned with the visual up regardless of the body’s actual posture. The agreement between the body and vision may help in the determination of which cue is the discrepant cue, leading to the gravity cue–the odd one out—being discounted. Our VRI-vulnerable group, with higher gravity weightings, would be more sensitive to detecting the visual/vestibular conflict in the MTT and to subsequently veto the gravity cue. This would then lead to a relative enhancement of the visual cues during the MTT, reflected as a higher gain and an increased likelihood of experiencing a VRI. The VRI-resistant individuals had a lower gravity weight, and were therefore likely less sensitive to the conflict and therefore less likely to veto the gravity cue (c.f., Weech et al. [14]).

### Conflict model

**Fig 5:**
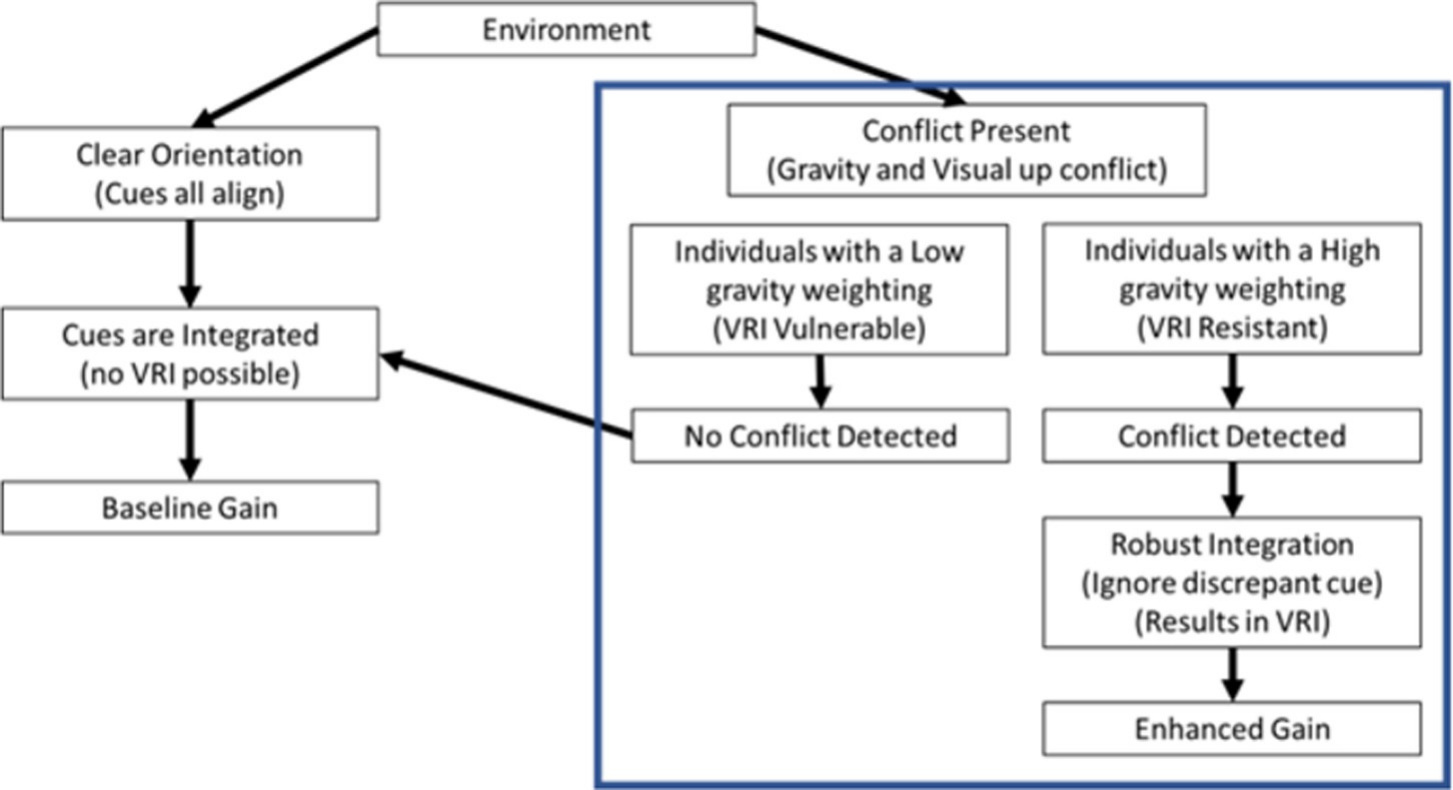
The effect of visual/vestibular conflict on visual gain. A model of how conflict between visual and vestibular cues to orientation might affect the gain of perceived motion. Within the blue box are two groups of participants, those with relativity high gravity weights (right), and those with relatively low gravity weights (left). The model indicates how the relative weights might alter perceived travel distance. The left side of the diagram indicates the situation when all cues are aligned.

In McManus and Harris [12], we proposed a “reweighting model” to explain the findings of the higher gain during a VRI. We proposed that when all the orientation cues in the environment (visual and non-visual) provide the same estimate of upright, participants should correctly feel upright and their visual weighting will be their baseline visual weight. In the absence of a detected conflict (VRI-resistant individuals), the visual weight would be unaffected. However, if a visual-vestibular conflict were detected the visual weighting under this model would be increased leading to an increase in the visual self-motion gain. Based on the findings reported here, we have now updated our model. The updated model purposes that in an environment with all of the cues aligned no sensory conflict is possible and so the sensory cues would be integrated normally resulting in no VRI and a baseline gain in a self-motion task. However, when a visual-vestibular conflict is present, a participant with a higher gravity weight would be more likely to detect the conflict leading to robust integration and the ignoring of the discrepant vestibular cue. Ignoring the gravity cue would then lead to a relative enhancement to the visual cue leading to a VRI and a higher visual gain. A participant with a low gravity weight on the other hand would not detect the visual-vestibular conflict and so would integrate the cues in the same way as an individual who is not experiencing a conflict. These VRI-resistant individuals would not experience an enhancement of the visual cues to motion and would have a gain similar to the baseline no-conflict condition. This model is drawn out in Fig 5.

## Conclusion

People’s vulnerability to experiencing a visual reorientation illusion correlates with their gain in the effectiveness of optic flow in evoking the sensation of self-motion: VRI-vulnerable people generally have higher gains while experiencing a VRI. Here we find that during a large sensory conflict, people who have a higher weighting of the gravity cue show an enhancement in their use of visual cues in generating self-motion. We hypothesise that this is due to robust integration of the cues leading to people ignoring the discrepant cue which results in a relative enhancement of the remaining cues. These results might help to explain previous findings on distance perception where perceived distance is compressed in the presence of a visual-vestibular conflict [37, 38]. Our findings add to the emerging body of literature revealing the widespread effects of sensory conflict on multiple aspects of perception [39, 40].

## Supporting information

S1 File All the supporting tables, figures, and data can be found in the S1 File document. (DOCX)
