# Supplementary material for: Enhancement of visual cues to self-motion during a visual/vestibular conflict
Source: PLoS One. 2023 Mar 15;18(3):e0282975. doi: 10.1371/journal.pone.0282975 (PMC10016722; doi:10.1371/journal.pone.0282975)
Supplement: S1 File — (DOCX) [file pone.0282975.s001.docx]

Supplementary Material For:

Enhancement of visual cues to self-motion during a visual/vestibular conflict

Meaghan McManus^1^ & Laurence R. Harris^2^

^1^Experimental Psychology, Justus Liebig University Giessen

^2^Centre for Vision Research, York University

Author For Correspondence ([meaghan.mcmanus@psychol.uni-giessen.de](mailto:meaghan.mcmanus@psychol.uni-giessen.de))
Address: Experimental Psychology, Justus-Liebig University Giessen, Otto-Behaghel-Strasse 10F, 35394 Giessen

# **VRI Measurements**

## **Method 1. Questionnaire following the move-to-target task**

At the end of each body posture during the move-to-target task (MTT), after the participant was instructed to remove the HMD, participants verbally answered a questionnaire to assess how their posture was perceived during the task as per Experiment 3 in McManus and Harris (2021).

After the participant completed their first posture in the MTT, the experimenter would ask the participant to remove the HMD and would read them the questionnaire read in full. In order to avoid unnecessary repetitions, for the 2^nd^ and 3^rd^ postures the experimenter omitted the first paragraph. The questionnaire in full was:

“I am going to describe three different ways you could have felt while you were in the virtual environment. It’s important that you think about how you felt while you were in the virtual environment, while you were stationary and while you were moving.

Due to the nature of virtual reality people can have different experiences. All of these are normal, and I am interested in how you felt while you were in the virtual environment. While you were in the virtual environment you might have felt one of three things, or possibly a combination of them. So, did you feel like you were:

1. looking up, and when moving, moving upwards. You might think of this lying down while flying upwards towards the sky

2. looking forwards, and when moving, moving forwards. You might think of this as moving similar to how you regularly move when not in VR, such as standing upright and moving forwards

3. looking down and when moving, moving downwards. You might think of this as lying down and looking over an edge down a cliff or maybe like falling

4. Some combination or other experience”

The order of options 1–3 were randomized each time they were asked however option 4 was always read last. After the experimenter was done reading the questionnaire the participant would verbally report which of the options, they felt best described their experience and the experimenter wrote down the participants response.

The participant listened to four possible options describing what they might have experienced in the VR environment. If the participants selected the option that would indicate they had experienced a VRI (they felt upright and looking and moving forward while they were actually tilted) they were grouped as having a VRI and given a score of “1”. If they selected one of the two options indicating they did not experience a VRI (they felt that they were tilted and looking and moving in a way that corresponded to their actual physical posture) they were grouped as “No VRI” and given a score of “-1”. The participant also had the option of “Other” if they felt the other options did not adequately describe their experience. These participants were grouped as “Other” and given a score of “0”.

## **Method 2. VRI Over Time**

The persistence of the VRI was measured over time. After completing all three postures of the MTT task the participants completed an additional ten trials of the MTT while supine. All participants completed the same set of five distances twice, with the final target distance being the target presented at 10m. After this, while still in the VR environment, participants were asked if their perceived orientation more closely matched the perception of “Standing upright, with your feet on a floor surface, looking forward” or “Lying down with your feet on a wall surface looking up”. They were then told that their perceived orientation would be measured for approximately 1½ minutes to get an idea of how it persists over time. They were told that if they felt closer to “Standing upright, with their feet on a floor surface, looking forward” they should click the left mouse button and hold it. If they felt their experience was closer to “Lying down with their feet on a wall surface looking up” they should click and hold the right mouse button. They were told to hold the button for as long as the feeling persisted and if at any point their perception should change, they should change which button they were holding. They were also instructed that it was possible their perception might not change and that was fine as well, so long as they were holding one of the two buttons at all times. The computer counted how many frames out of 5,000 frames at 90 frames a second (approximately 1.5 minutes) the left mouse button was pressed. An “upright” response for a total of 2,500 frames (half the time) or more was classified as “VRI” and the participant was given a score of “+1”. Zero frames was scored as “No VRI”, score “-1”. If the VRI button was pushed for between 1 and 2,400 frames they were grouped as “Other”, score “0”.

## **Method 3. VRI Verbal Report**

After completing the left-side-down posture for the OChaRT, participants were asked to describe how they felt generally during the task, with regards to their orientation. If their verbal report more closely matched an upright experience they were grouped as “VRI”, score “+1”. If it more closely matched lying down, they were grouped as “No VRI”, score “-1”. A response closer to “unsure”, “sometimes”, or “confused” were grouped as “Other”, score “0”.

# **VRI Group Decision**

During the OChaRT, the visual background changed every trial so a VRI could not be determined at the end of each body posture as accurately as the MTT. As well, the VRI likelihood while left-side-down might not be the same as the likelihood of experiencing a VRI while supine or prone. Therefore, in order to determine the VRI groups the scores for each of the different VRI measures was summed. If the sum was positive then the participant was grouped as being “VRI-vulnerable” and was considered a “VRI” person, if the sum was negative the participant was grouped as “VRI-resistant” and was considered a “no-VRI” person, and if the sum was “0” the person was grouped as “Other”. Additionally, one participants VRI Over Time task was not recorded. Their summation was then performed over only the other measures and excluded that one.

# **Breakdown of the VRI Measures**

See Table 1 Supplementary for a breakdown of the VRI results based on each of the VRI measures.

Table 1 Supplementary. The table shows the percentage of participants who were grouped as having a VRI, not having a VRI, or “Other” on our three VRI measures, as well as the results of the overall VRI group classification. The “MTT Supine” and “MTT Prone” rows show the results of the questionnaire following the MTT task while supine or prone. The “Time” row shows the results of the “Over Time” method, and the “OChaRT LSD” refers to the response given during the “VRI Verbal Report” following the left side down posture of the OChaRT. The “VRI Group” row shows the result of the overall “VRI Group Decision” summation based on the results of the other VRI measures. The numbers in the brackets are the number of participants in each group.

| VRI Measure | VRI % | No VRI % | Other % |
| --- | --- | --- | --- |
| MTT Supine | 46.34 (19) | 31.71 (13) | 21.95 (9) |
| MTT Prone | 36.59 (15) | 26.83 (11) | 36.59 (15) |
| Time | 51.22 (21) | 31.71 (13) | 14.63 (6) |
| OChaRT LSD | 24.39 (10) | 63.41 (26) | 12.20 (5) |
| VRI Group | 51.22 (21) | 39.02 (16) | 9.76 (4) |

# **MTT Outlier Analyses and Distribution of Data**

Each participant had 147 data points (5 distances x 10 repetitions x 3 postures, however due to an error the experiment finished one trial early so each posture is missing one trial).

Three participants were removed from the MTT analysis as they either could not perform the task or were not able to discriminate between the different distances. Then a within subjects outlier analysis was performed, followed by a between subjects outlier analysis.

Firstly, the raw distance traveled data was checked for mistrials. A mistrial might occur if the participant accidently hit the stop button right after hitting the start button. Mistrials were determined as any stop distance less than 0.5 m as the optic flow was presented at 9.8m/s/s where a distance of less than half a meter would indicate the participant pressed the stop button after around 1/20th of a second after starting. This resulted in the removal of 14/5586 data points. Following this, outliers were removed. A data point was removed if it was ±2SD away from the mean. This resulted in the removal of 218/5572 points or 3.9% of the data. The average and standard deviation was then determined for each participant and for each posture and distance resulting in 570 data points (5 distances x 3 postures x 38 participants).

The between subjects analysis outlier analysis was then run where, for each target distance in each posture, data were removed if they fell outside ±2.5 standard deviations from the mean. See Figure 1 Supplementary for the distribution of the data.

Using this data the residuals were plotted in QQ plots and boxplots. It was revealed that some additional data points were still significant outliers. For example, with the boxplots there were still data that fell 3 boxes or more away from the upper and lower hinges (the hinges for the central 50% of the data around the median) indicating they fell outside of 99% of the rest of the distribution (the distribution of data following the ±2 standard deviation removal). These data points were removed resulting in a total of 35 out of 570 data points being removed (6.14%).

##### Of the three participants who were removed for not being able to discriminate distances, one was from the VRI-resistant group, one was in the VRI group, and one was from the “Other” group.


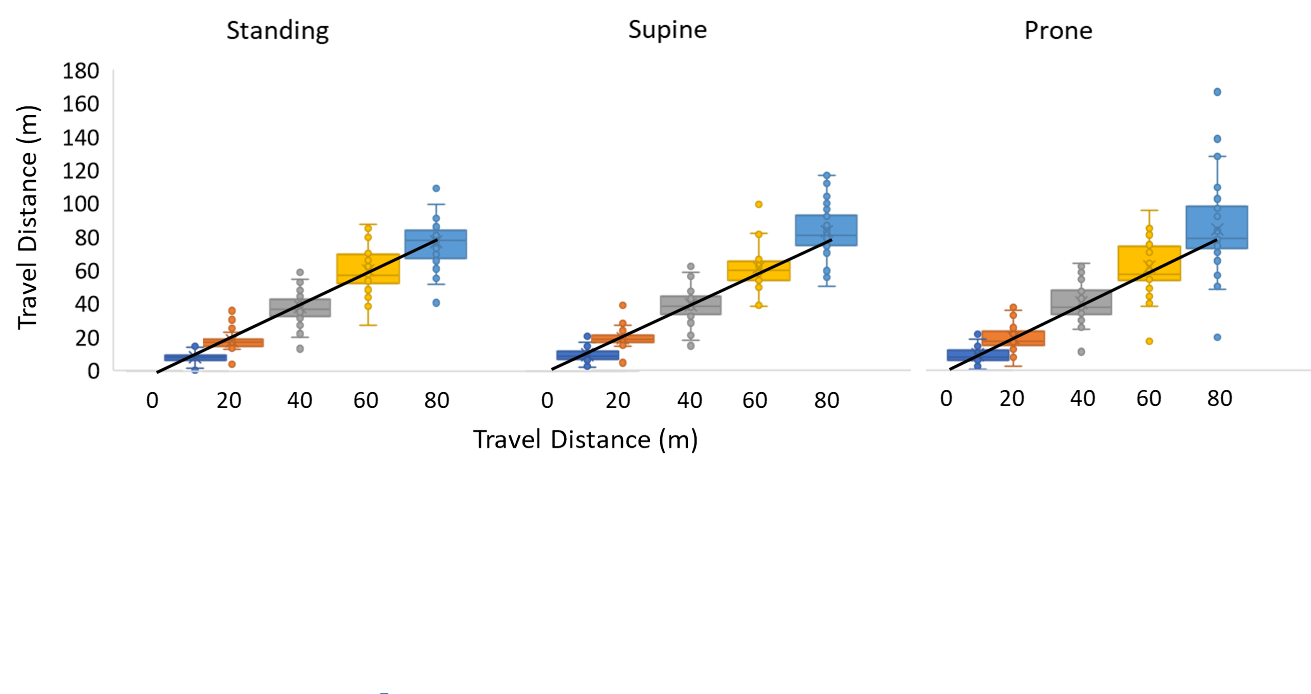


Figure 1 Supplementary. The distribution of the data for each target distance for each posture are plotted in boxplots. A boxplot divides the data into quantiles where the box represents a range of one quartile and the line represents the median. The “whiskers” on either end define 1.5 quartiles from the edge of the box. Mild outliers fall outside of that range with extreme outliers falling 3 quartiles from the edge of the box. The solid line represents perfect performance.

# **OCHaRT Outlier Analysis**

Before fitting the OChaRT data to the model, outliers were removed. For each of visual angles the average angle and standard deviation was found. Any value that fell outside of 2 standard deviations from the mean was removed. This resulted in 22 out of 410 (5.37%) OCHaRT data points being removed. For any participant who had missing data, when their weightings were fit to the model that visual angle for that posture was simply excluded from the fit. However, after the outlier analysis one participant had only 3 data points left, with no data from their lying posture. Because of this no reliable estimate of the PU could be obtained and they were removed from the analysis resulting in a total of 26 out of 410 (6.34%) data points being removed.
